# Aroma precursors of Grignolino grapes (*Vitis vinifera L.*) and their modulation by vintage in a climate change scenario

**DOI:** 10.3389/fpls.2023.1179111

**Published:** 2023-08-04

**Authors:** Andriani Asproudi, Federica Bonello, Vasiliki Ragkousi, Silvia Gianotti, Maurizio Petrozziello

**Affiliations:** ^1^ Research centre for Viticulture and Enology, Council for Agricultural Research and Economics (CREA), Asti, Italy; ^2^ Associazione Monferace, Alessandria, Italy; ^3^ Wine Consulting Mario Ronco, Asti, Italy

**Keywords:** C_13_-norisoprenoids, Benzenoids, Terpenoids, Grignolino, seasonal stress conditions, Hilly viticulture, old vineyards

## Abstract

Current climatic conditions may cause significant changes in grapevine phenology and maturity dynamics linked often with changes to ecoclimatic indicators. The influence exerted by different meteorological conditions during four consecutive years on the aromatic potential of Grignolino grapes was investigated for the first time. The samples were collected from three vineyards characterized by different microclimatic conditions mainly related to the vineyard exposure and by a different age of the plants. Important differences as far as temperature and rainfall patterns are concerned during ripening were observed among the 4 years. Grape responses to abiotic stress, with particular emphasis on aromatic precursors, were evaluated using gas chromatography coupled to mass spectrometry. The results highlighted significant differences among the vintages for each vineyard in terms of the berry weight and the aromatic precursor concentration. For the grapes of the younger-vine vineyard, the content of aroma compounds showed a different variability among the vintages if compared to the old-vine vineyards. Optimal conditions in terms of temperature and rainfall during the green phase followed by a warm and dry post-veraison period until harvest favored all classes of compounds especially terpenoids mainly in the grapes of the old vines. High-temperature (>30°C) and low-rainfall pattern before veraison led to high benzenoid contents and increased differences among vineyards such as berry weight, whereas cooler conditions favored the terpenoid levels in grapes from southeast-oriented vineyards. In a hilly environment, lack of rainfall and high temperature that lately characterize the second part of berry development seem to favor the grape quality of Grignolino, a cultivar of medium-late ripening, by limiting the differences on bunch ripening, allowing a greater accumulation of secondary metabolites but maintaining at the same time an optimum balance sugar/acidity.

## 1 Introduction

Most wine-producing regions around the world suffer from the effects of climate change. In the temperate zones, large fluctuations of the main climatic parameters are observed over the years, and above all, a gradual and irreversible tendency towards an increase in average seasonal temperatures and a significant decrease in rainfall associated to a different distribution during the grape ripening period. Grapevine cultivation for winemaking is highly dependent on climate fluctuations that exert profound effects on vine phenology and grape composition. Global climate change directly modifies mainly abiotic factors such as temperature and both water availability and distribution during the grapevine growth cycle (Jackson and Lombard, 1993; Fraga et al., 2018; Rienth et al., 2021). Additionally, climate change can cause shifts in phenological stages, resulting in alterations to the optimal photoperiod. This, in turn, affects the rate of photosynthesis and the phenological stages of the grapevine, ultimately influencing grape composition.

The increase in average and extreme temperatures of summer months, as a major consequence of climate change, as well as changes of rainfall patterns during the ripening phase, leads not only to a higher concentration of sugar and a general change of the acidic profile of grapes, but also to the modification of secondary metabolite profiles (De Orduna, 2010; Van Leeuwen and Darriet, 2016; Asproudi et al., 2018). Therefore, in several viticulture areas, ripening occurs when both color and aroma profile can be adversely affected (Mori et al., 2007; Asproudi et al., 2016), which, in wine, is translated to a loss of typicity and terroir expression (van Leeuwen et al., 2020). Owing to climate change, a mismatch is also noted between the different parameters of technological, phenolic, and aromatic quality as they are regulated by different biosynthetic pathways and therefore are influenced differently.

Sunlight radiation has been suggested to promote grape ripeness, modulating the metabolic profile of berries, and activating the synthesis and accumulation of diverse compounds in the skin of berries, including sugars, organic acids, amino acids, and phenylpropanoids (Carbonell-Bejerano et al., 2014), although it was difficult to separate the combined effect with temperature.

High sun irradiation and high temperatures after veraison are reported to be factors that greatly affect the ripening of grapes (Bergqvist et al., 2001) and can lead to an anticipation of the ripening period earlier during the warmest part of the season. Conversely, mild temperatures during spring and ripening and late rainfall events during veraison might cause a delay in the ripening process, generating grapes with low pH, high total acidity, and high varietal aroma potential, as previously mentioned (Webb et al., 2012).

Several other parameters such as the age and the exposure of vines as well the altitude of vineyards may affect the response of plants to climate change. Younger vines, for instance, seem to be more sensitive to water stress conditions, due to less developed root systems, whereas vine age may also affect phenology and gas exchange parameters (Riffle et al., 2021). Young vines have been reported to show lower photosynthesis (Zufferey and Maigre, 2007), stomatal conductance, and transpiration compared to old vines. As a consequence, a slower berry formation but quicker berry ripening may be noted, and optimal technological ripening parameters are reached earlier than in old vines (Bou Nader et al., 2019).

The effects of high sun radiation and temperatures on secondary metabolism control have been characterized for phenolics, mostly in artificial growing conditions, while little is known with respect to aromas; a decrease in the skin/pulp ratio, with possible effect on the aromatic potential, was mentioned (Pérez-Álvarez et al., 2021). The concept of grape and wine quality is inextricably linked to secondary metabolites. The color, aroma, and flavor of the wine, as well as their stability over time, depend strictly on the concentration of these compounds present in the grapes and their transformation during the winemaking process. These transformations are well known and can include oxidation, reduction, enzymatic reactions, and chemical reactions between compounds, such as esterification, hydrolysis, and polymerization. These changes can significantly impact the sensory profile and overall quality of the final wine product. Conversely, a relatively limited number of studies have dealt with the effect of climate and microclimate factors on the volatile composition of grapes and on aroma precursors, although much of the perceived quality of a wine by the consumer depends closely on the contribution to aroma of these molecules.

Some studies evidence the probable effect of sunlight conditions on some aroma compounds (e.g., terpenes, alcohols, and aldehydes) since they seem implicated in the protection of grapes against photooxidation (He et al., 2020). Moreover, recent research indicated that microclimate conditions, which varied according to the vintage, may affect the timing of the peak concentration of norisoprenoid precursors and their final content in Nebbiolo berries whereas high temperature during the last stages of grape ripening evidently led to a decrease of the total norisoprenoids (Asproudi et al., 2016). Previous research on other cultivars instead, implicating modified light condition in the fruit zone (leaf management), showed an increase of norisoprenoids in the grapes of Riesling, Pinot, noir, and other cultivars (Feng et al., 2015; Yuan et al., 2015; Alem et al., 2019).

Most volatile benzenoids are generated from phenylalanine through the shikimate acid pathway and it has been demonstrated that environmental stresses can activate this pathway and further promote the accumulation of secondary metabolites (Zhang et al., 2012).

Certainly, climate fluctuations among seasons may lead to relevant quality differences between wines produced from grapes of the same cultivar (Crespo et al., 2018). A cultivar maintains typical characteristics if cultivated in different regions, but the aromatic potential of grapes and wine strongly depends on meteorological factors such as rain level and temperature fluctuations during the berry development and the ripening period (Crespo et al., 2018). Thus, in the context of significant changes in climate and increased stress conditions, further studies should analyze the specific consequences of these multifactorial phenomena on the aromatic and flavor components of wines and their aging potential (Drappier et al., 2016; Wu et al., 2019).

Lately, winemakers are looking for vineyards that face north, as well as those at higher elevations, while southwest-facing vineyards have been seen to be optimum in case of stress, light, and temperature conditions (Hannah et al., 2013). For centuries, some of the world’s greatest vineyards in the Northern Hemisphere were planted on hillsides, with suitable soils, facing south or southeast, where they would receive the most sun and warmth, allowing grapes to fully ripen (Santos et al., 2020). The same formula governed the placement of Grignolino vineyards since this *cv* often displays non-uniform ripening and berries in the same bunch are often of very different color, due to the uneven ripening.

Grignolino is an Italian autochthonous cultivar of high interest for the local (North Italy) and international wine market. Despite being a native cultivar closely linked to its original territory (Piedmont), it is also grown in other Italian regions as well as in California and the United States outside of Italy (Robinson et al., 2013). This grape has probably been cultivated in the Monferrato hills, since the XIII century (Mainardi, 2003) and nowadays represents the historical and oenological identity of the Monferrato in Piedmont. This territory, included in the World Heritage List designated by UNESCO, is famous for its strong vocation to produce high-quality wines from several autochthonous grape varieties.

More specifically, this region, is a rainfed hilly vine-growing area in Piedmont in which current climatic conditions caused significant changes in grapevine phenology and maturity dynamics, depending on the variety. Recent research, indeed, investigated the influence of increasing temperature and Huglin index in anticipating the harvest period, particularly the harvest beginning, mentioning that it was highly significant for all the considered varieties and vineyards in the Monferrato area (Bagagiolo et al., 2021).

Grignolino grapes, the subject of this study, are obtained from vines cultivated, according to the guidelines for the production of “Monferace” wine, in the Aleramic Monferrato region, which is bounded by the Po and Tanaro rivers in the heart of Piedmont. The vineyards are exclusively hilly with optimal sun exposure to ensure proper grape ripening. The soils must be calcareous–silt–clayey, in various combinations, with the possibility of naturally occurring sandy sediment. No vine forcing is permitted; only emergency irrigation is allowed (Monferace a Grignolino of Excellence, 2023). These wines require many years to reach a balance between the aromatic complexity and pronounced astringency and acidity. From an aromatic point of view, Grignolino is a cultivar with a neutral character characterized by significant concentrations of glycosylated aroma compounds, particularly terpenols, norisoprenoids, and benzenoids, which may release their potentially odoriferous free aglycons during winemaking or storage, contributing to define the final complex and distinctive aroma of the wine. Some recent research highlighted that aroma glycoside conjugates can also influence taste properties of the wine, such as the aromatic persistence (Parker et al., 2017; Liang et al., 2022). The concentration of grapes in aromatic precursors may thus significantly influence the fragrance of long-aged wines. Furthermore, the amount and type of glycosylated aromas present in a wine are influenced by multiple factors, including the weather conditions during berry development and ripening (Ferreira and Lopez, 2019; Caffrey and Ebeler, 2021). It is therefore essential to evaluate the impact of climate change on grape aroma precursors in order to adapt new strategies aimed to maintain high-quality productions.

This research study willed to add new knowledge about this important Italian variety to face the challenge of climate crisis, and to comprehend when, how, and which meteorological variables may affect the various chemical classes of compounds that determine the wine aroma. To assess the influence of different meteorological conditions among years on the aroma potential of Grignolino grapes, solid-phase extraction (SPE) and subsequent enzymatic hydrolysis were the analytical strategies used, respectively, to isolate and release the glycosylated grape compounds prior to their determination *via* gas chromatography–mass spectrometry (GC–MS).

## 2 Materials and methods

### 2.1 Vineyard sites and grape technological composition

Data presented in this article concern four consecutive years, over the 2019, 2020, 2021, and 2022 growing seasons. Grape samples of Grignolino *cv* were collected in three different vineyards, located near Vignale Monferrato (45°0’45’’ N, 8°23’51’’ E, Piedmont, Italy), one with a medium vine age of 20 years (young) and two with 50-year-old vines (old). Plants were grafted onto 1103 Paulsen, the training system used is Guyot, with an elevation of approximately 300 m a.s.l. and a plant density from 4,500 to 5,000 plants/hectare all planted in a hilly area. The three vineyards were characterized by different microclimatic conditions mainly related to different aspects and vine ages: one old-vine vineyard was southeast oriented (Old SE), whereas the second old-vine and the younger-vine vineyards were southwest oriented (Old SW and Young SW). During this study, no emergency irrigation was carried out.

Three blocks for each vineyard were identified as representative and used as biological replicates The sampling of Grignolino grapes was carried out for four consecutive years at ripening. The harvesting time was established according to the strategy adopted by each wine company for the specific year. Approximately 15 clusters for each biological replicate were harvested manually. For each replicate, 500 berries were collected with their pedicels randomly from different sides of the bunches, both shady and sunlit, to prepare grape extracts for aroma and phenolic compound measurements. The analysis of the main physicochemical parameters, namely, berry weight, titratable acidity (TA), and total soluble solids (TSS), were also carried out at ripening (Compendium of international methods of wine and must analysis-OIV, 2020).

Extraction of the polyphenolic fraction from the grapes was performed according to previous works (Di Stefano and Cravero, 1991). Total polyphenol content (TPI, total polyphenol index) was determined using the Folin–Ciocalteu method, whereas the determination of anthocyanins (TAI, total anthocyanin index) was carried out spectrophotometrically as described by Di Stefano and co-workers with some revision (Di Stefano and Guidoni, 1989; Asproudi et al., 2018).

### 2.2 Climate and meteorological assessments

The meteorological data, measured in 2019, 2020, 2021 and 2022, were provided by a weather station near the vineyards (Vignale Monferrato) equipped with a thermohydrometer and a rain gauge (**Table 1**). The station is managed by the Meteo hydrographic Monitoring Network of Arpa Piemonte (Piedmont Environmental Protection Agency). The average values of the period 2009–2018 were obtained from data collected from the same weather station. The mean of maximum temperatures (Tmax mean), the mean of minimum temperatures (Tmin mean), the average of the monthly maximum temperature values, the number of days with temperatures higher than 35°C (T > 35°C), the total rainfall, and the number of rainy days with rainfall of more than 1 mm (RD ≥ 1) are shown in **Table 1**.

**Table 1:** Meteorological characterization of the four seasons (the values are calculated both for the entire year “a” and for the grapevine vegetative period: April–October “b”).

a) January to December	Tmax	Tmin	Tmax-M	Total rainfall	GDD 10°	Days T>30°C	Rainy days
**Years**	**°C**	**°C**	**°C**	**mm**	**°C**	**number**	**number**
2019	20.9	**11.8**	27.1	773	–	**56**	72
2020	20.6	11.5	**26.5**	629	–	45	68
2021	20.6	**11.0**	**26.6**	**507**	**-**	45	**53**
2022	22.2	**12.6**	**28.7**	**349**	**-**	**76**	**37**
*Averages 2009–2018*	*20.9*	*11.4*	*27.5*	*673*	*-*	*53*	*66*
*b)* April to October	Tmax	Tmin	Tmax-M	Total rainfall	GDD 10°	Days T>30°C	Rainy days
**Years**	**°C**	**°C**	**°C**	**mm**	**°C**	**number**	**number**
2019	**24.6**	14.7	30.5	443	2,069	**56**	**42**
2020	24.8	14.4	**29.0**	**509**	2,046	45	**45**
2021	**24.5**	14.1	**29.9**	**329**	**2,005**	45	**26**
2022	25.4	14.7	**32.3**	**216**	**2,407**	**76**	**24**
*Averages 2009–2018*	*25.1*	*14.4*	*30.7*	*388*	*2,102*	*53*	*37*

Data reported were recorded by the Vignale Monferrato meteorological station after reprocessing based on measures collected by the Arpa Piemonte Meteor Hydrographic Monitoring Network.

Tmax: average daily maximum air temperature (°C); Tmin: average daily minimum air temperature (°C); Tmax-M average monthly maximum air temperature; Total rainfall: annual total precipitation (mm); GDD 10°: Growing Degree Days using a lower threshold of 10°C; days T>30°C: number of days with maximum temperatures above 30°C; Rainy days: Days with precipitation over 1 mm. Bold values denote statistically significant differences against average values calculated between 2009 and 2018 (one-sample z-test/two-tailed test; p < 0.05).

Cumulative growing degree days (GDD) were computed as the sum of the average daily temperature above a base temperature of 10°C (Amerine and Winkler, 1944). GDD were calculated from the 1 April to 30 October.

Furthermore, to better characterize each season, two periods were considered and the sum of rain, GDD, T > 35°C, and T in the range between 25 and 35°C were assessed during the first pre-veraison period (April–August) and after veraison until harvest (August–September).

### 2.3 Extraction and determination of aroma precursors from grapes

#### 2.3.1 Extraction of bounded aroma compounds from grapes

A total of 100 berries of Grignolino, previously weighted and stripped of seeds, were homogenized (Di Stefano et al., 1998). The suspension was then centrifuged (4000*g* for 15 min) and the supernatant was transferred to a 300-ml volumetric flask, washed, and brought up to volume with tartaric acid buffer (pH = 3.0, 0.04 M). Three replicates of all samples were analyzed. The isolation of grape heterosides was performed as previously reported for a grape and wine matrix (Mateo et al., 1997; Asproudi et al., 2016; Asproudi et al., 2018) after appropriate modifications for better extraction of target compounds from Grignolino grape extracts. Briefly, 250 ml of extract was passed through a 5-g C18 End Capped cartridge (Biotage AB, Uppsala, Sweden) previously activated with methanol and distilled water in sequence. After washing (water and dichloromethane), the glycosides were recovered with 25 ml of methanol (Sigma Aldrich Co., St. Louis, MO, USA). C18 sorbents were indicated to be more suitable for selective extraction of less polar precursors such as terpenic and norisoprenoid precursors. Furthermore, the same standardized procedure was applied for all samples to obtain reliable comparison results between vineyards and vintages (Jesús Ibarz et al., 2006; Liu et al., 2017).

#### 2.3.2 Hydrolysis of glycosides by exogenous enzyme

The hydrolysis of glycosides by exogenous enzyme was carried as previously reported (Mateo et al., 1997; Asproudi et al., 2016; Asproudi et al., 2018). Briefly, the methanolic phase underwent evaporation until dryness was achieved, employing a rotary evaporator under reduced pressure keeping bath temperature and rotation speed of the flask constant and equal for all samples. Subsequently, the residue was dissolved in 5 ml of citrate-phosphate buffer, adjusted to a pH of 5.0. Then, the enzymatic hydrolysis was carried out with Pectinol (Genencor, Palo Alto, CA, USA) with glycosidase side activity at 40°C for 24 h, to obtain free aglycon compounds. After hydrolysis, 0.25 ml of 2-octanol (50 mg/L), used as internal standard, was added and the extract passed through a 1-g C18-RP cartridge previously activated to isolate the aglycons. After a water wash and drying step, the aglycons were eluted with dichloromethane and collected in a 25-ml Erlenmeyer flask. The organic layer was dried using anhydrous Na_2_SO_4_, transferred into a distillation flask, and reduced to a small volume (approximately 200 µl) at room temperature. The sample was stored at −20°C until the analysis by GC–MS.

#### 2.3.3 Gas chromatography–mass spectrometry determinations

GC–MS analysis was carried out by an Agilent 7890A gas chromatograph, equipped with an Agilent 5975C Mass Selective Detector (Agilent Technologies, Palo Alto, CA, USA). The samples (2 μl of dichloromethane extract) were manually injected into the injector at 250°C in split less mode. The separation was achieved using a Zebron ZB-WAX, Column 60 m × 0.25 mm × 0.25 μm (Phenomenex, Torrance, CA, USA). The oven temperature was held at 45°C for 2 min, then raised to 60°C at a rate of 30°C/min, from 60 to 230°C at a rate of 2°C/min, and held at 230°C for 20 min. Helium was the carrier gas and the column flow was maintained at 1.2 ml min^−1^. The transfer line was set at 230°C. The ionization voltage was 70 eV, the quadrupole was set at 230°C, and the source was set at 250°C. The acquisition of mass spectra for the analysis of compounds was carried out in total ion current mode (TIC) and a 29–300 m/z range was recorded. To identify the volatile compounds, mass spectra and retention indices of authentic standards were compared to those of the analytes. In cases where an authentic standard was not available, recorded spectra and LRI values were compared to the NIST14 and WILEY275 databases, as well as the gas chromatographic retention indices reported in the literature (see **Supplementary Table 1**). The quantification was performed by comparing the areas of the chromatographic peaks to that of the internal standard (IS), 2-octanol, and the compound concentrations were calculated as equivalents of 2-octanol, which was used as the IS. Particular attention was given to the composition of the grapes in terpenoid, C_13_-norisoprenoid, and benzenoid compounds. The results were expressed both as μg/kg of berries, as frequently reported in the literature, and as μg/100 berries to prevent the influence of the different berry size on the result interpretation.

#### 2.4.5 Statistical analysis

All statistical analyses were carried out using XLSTAT-Pro (Data Analysis and Statistical Solution for Microsoft Excel, Addinsoft, Paris, France 2017). To generally examine the effects of the vintage (year) and vineyard as well vineyard and vintage interaction (Vineyard *Year) on berry physical, chemical, polyphenolic, and aromatic composition, data were subjected to three two-way ANOVAs and differences among means were assessed according to the least significant difference from Tukey’s test with a confidence interval of 95% (*p* < 0.05). To obtain more information, the relationship between meteorological parameters, vineyards, and vintages, with the chemical classes of the volatile hydrolytically released compounds quantified in Grignolino grape berries, was assessed with a two-principal component analysis (PCA) multivariate approach using XLSTAT software.

## 3 Results and discussion

### 3.1 Meteorological assessment

**Figure 1:**
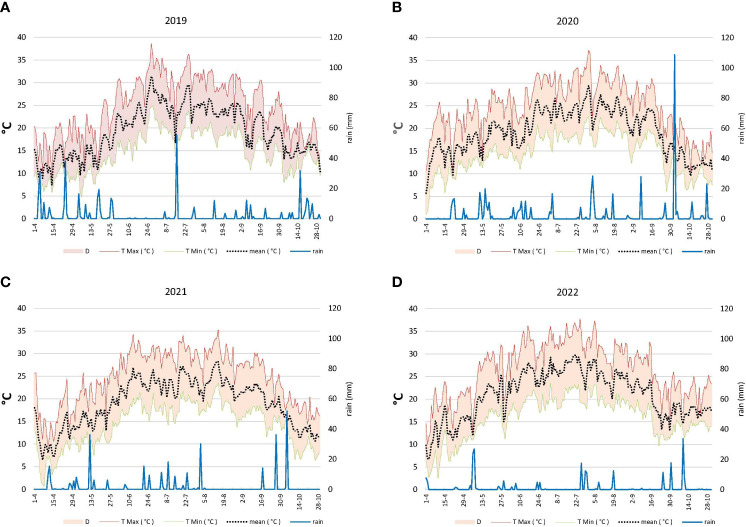
**(A)** Meteorological pattern during 2019. T: temperature; D: differences between T max and T min. **(B)** Meteorological pattern during 2020. T: temperature; D: differences between T max and T min. **(C)** Meteorological pattern during 2021. T: temperature; D: differences between T max and T min. **(D)** Meteorological pattern during 2022. T: temperature; D: differences between T max and T min.

The four considered vintages presented some peculiarities from a meteorological point of view. Season 2019, as shown in **Figure 1A** was characterized by maximum temperatures similar to the average values monitored over the previous decade (**Table 1**), whereas the days with temperatures above 30°C were slightly higher than those of the reference period taken into consideration. In the Vignale area, the 2019 annual rainfall was more abundant compared to the subsequent years but did not show a statistically significant difference when compared to the previous 10 years. The early spring period that coincides with the green phase of the berry development was characterized by frequent rain events and average temperatures that rarely exceeded 20°C followed by a hot (>30°C) and dry pre-veraison period. Concurrently to the veraison, a strong rainfall was registered, whereas after that, an equal distribution of light rainfall until harvest was observed.

2020 was the cooler year if compared to the others (**Figure 1B**), with maximum temperatures lower than the mean values registered in the decade 2009–2018 that rarely exceeded 30°C. Even if the annual total rainfall was similar to the reference period, rainfall was much more concentrated from April to October, that is, in the period of berry development and ripening, especially in two phases before veraison and close to harvest.

On the other hand, the 2021 (**Figure 1C**) season was characterized by maximum temperatures, referring to the April–October period, lower than those of the decade 2009–2018; number of rainy days and total rainfall were significantly lower too. A regular distribution of rainfall during the green phase and until veraison can be noticed followed by a warm and dry post-veraison period until harvest.

For 2022 vintage (**Figure 1D**) instead, a series of extreme conditions occurred in terms of both temperature and rainfall. Monthly Tmax, heat accumulation (GDD), and particularly the number of days with maximum temperatures above 30°C (76 days) were significantly above the average values registered for the previous decade, while often temperature exceeds 35°C during the veraison time. Conversely, values of rainy days and especially total rainfall were decidedly lower during ripening with respect to the previous years of study and the reference decade, which denotes scarce water availability more specifically in the green phase of berry development. Meteorological conditions observed for 2022 brought an anticipation of approximately 10 days of both veraison (25/30 July) and ripening (5/10 September).

### 3.2 Grape technological parameters

Grignolino grapes showed an adequate and uniform bunch ripening in the 4 years of study. The age and exposure of the vineyards did not appear to have an impact on the timing of phenological phases, such as veraison and maturity, that occurred in the same period in all vineyards for each year. Grapes harvested over the 4 years of study in the three vineyards reached technological maturity and were harvested during the third week of September for the first 3 years, whereas for 2022, a 10-day advance shift of the harvest was registered. For 2019 and especially for 2022 vintage, mean berry weight was significantly lower at harvest with respect to the two vintages 2020 and 2021 (**Table 2**). Moreover, the data regarding the berry composition and main polyphenolic parameters showed that the average values among years were similar as regards TA and pH, with slightly higher °Brix values for the 2022 season and lower polyphenolic levels in 2020. On average, vineyard Old SE had significantly higher contents in TAI and TPI when results are expressed per berry, and also had considerably higher berry weight while Young SW berries tend to present the lowest values as regards technological parameters.

**Table 2:** Principal chemical and physical analysis on Grignolino berries harvested in vintages 2019, 2020, 2021, and 2022.

	Year (Y)				Vineyards (V)			ANOVA		
	2019	2020	2021	2022	Old SE	Old SW	Young SW	sig. Y	sig. V	sig. Y*V
**Berry weight (g)**	1.34 **b**	1.80 **a**	1.74 **a**	1.18 **c**	1.70 **a**	1.43 **b**	1.41 **b**	***	***	**
**TSS (° Brix)**	25.1 **b**	24.0 **b**	24.5 **b**	26.3 **a**	25.8 **a**	24.3 **b**	25.0 **ab**	**	*	ns
**TA (g/L)**	6.9	6.5	6.9	6.4	6.8	6.7	6.5	ns	ns	ns
**pH**	3.25	3.30	3.18	3.25	3.24	3.25	3.24	ns	ns	ns
**TPI (mg/berry)**	2.9 **a**	2.4 **b**	2.6 **a**	1.7 c	2.7 **a**	2.2 **b**	2.2 **b**	***	***	***
**TPI (mg/kg)**	1.690 **a**	1.317 **b**	1.529 **a**	1.478 **a**	1.697 **a**	1.548 **b**	1.566 **b**	**	**	***
**TAI (mg/berry)**	0.6 **a**	0.4 **b**	0.6 **a**	0.4 **b**	0.6 **a**	0.5 **b**	0.4 **c**	***	***	***
**TAI (mg/kg)**	425 **a**	225 **b**	351 **a**	364 **a**	356 **a**	350 **a**	317 **b**	***	***	**

TSS, total soluble solids; TA, titratable acidity; TPI, total polyphenol index measured in berry skins as mg/kg and mg/berry; TAI, total anthocyanin index measured in berry skins as mg/kg and mg/berry. Data were subjected to the analysis of variance (ANOVA) and post-hoc Tukey test. ANOVA significance, ns (not significant): p-value > 0.05, ⁠*0.01 ≤ p-value < 0.05, ⁠**0.001 ≤ p-value < 0.01, ⁠***p-value < 0.001. Different letters indicate different least square means.

### 3.3 Aroma precursors of Grignolino berries

The main hydrolytically released compounds detected in the berries of Grignolino can be arranged into four groups, namely, terpenoids, C_13_-norisoprenoids, benzenoids, and C_6_ aldehydes and alcohols (see **Supplementary Table 1**), in amounts consistent with the non-aromatic character of this cv. The most representative compounds among terpenoids were geraniol, p-ment-1-ene-7,8-diol, and geranic acid; among C_13_-norisoprenoids, 3-oxo-α-ionol, 3-OH-β-damascone, and 3-hydroxy-7,8-dihydro-β-ionol; and among benzenoids, benzyl alcohol and phenylethyl alcohol. The primary C_6_ aldehydes and alcohols identified were 1-hexanol (see **Supplementary Tables 2**, **3**).

The first results of ANOVA carried out on the average total values of the aroma compounds, to globally examine the effects of the vintage (year) and vineyards, are shown in **Table 3**. From this first analysis, we detected that both season and vineyard exerted a significant influence on most of the variables (classes of aroma precursors) studied. The composition of the grapes collected from the different vineyards over the 4 years indeed showed significant differences among years for almost all classes of precursors. Considering the average content values measured in the grapes of the three vineyards for each year of study, we can note higher contents of C_13_-norisoprenoids, benzenoids, and C_6_ aldehydes and alcohols for 2022 when results are expressed as μg per kg of berries and significantly higher values of terpenoids and C_13_-norisoprenoids per berry for 2021. In 2021, the terpenoid amounts were mainly significantly higher than in the other years. 2020 presents the lowest content of precursors especially in terpenoids and benzenoids. Benzenoids, instead, were the most represented compounds in the berries of Grignolino for 2019 and 2022, whereas terpenoids and benzenoids were the most represented compounds for 2021 vintage.

**Table 3:** Average values of each chemical aroma family measured in the three vineyards (Old SW, Old SE, and Young SE) for each vintage year (2019, 2020, 2021, and 2022).

		Year (Y)				Vineyards (V)			ANOVA		
		2019	2020	2021	2022	Old SW	Old SE	Young SW	sig. Y	sig. V	sig. (Y*V)
**Terpenoids**	µg/100 berries	63 **b**	53 **b**	112 **a**	70 **b**	69 **b**	105 **a**	49 **c**	***	***	ns
	µg/kg of berries	464 **b**	319 **c**	634 **a**	585 **ab**	496 **b**	607**a**	398 **b**	***	**	ns
**C_13_-Norisoprenoids**	µg/100 berries	27 **b**	32 **b**	50 **a**	40 **ab**	33 **b**	49 **a**	29 **b**	**	**	ns
	µg/kg of berries	197 **b**	193 **b**	284 **ab**	344 **a**	239 **a**	284 **a**	240 **a**	**	ns	ns
**Benzenoids**	µg/100 berries	85 **b**	54 **c**	118 **a**	128 **a**	99 **b**	128 **a**	62 **c**	***	***	**
	µg/kg of berries	607 **b**	330 **c**	663 **b**	1.090 **a**	708 **a**	768 **a**	541 **b**	***	**	***
**C_6_ aldehydes and alcohols**	µg/100 berries	12	15	15	16	14 **b**	18 **a**	11 **b**	ns	**	*
	µg/kg of berries	86 **b**	88 **b**	79 **b**	142 **a**	99 **a**	106 **a**	91 **a**	**	ns	ns

Data were subjected to the analysis of variance (ANOVA) and post-hoc Tukey test. Means followed by a different letter are significantly different. ANOVA significance, ns (not significant): p-value > 0.05, ⁠*0.01 ≤ p-value < 0.05, ⁠**0.001 ≤ p-value < 0.01, ⁠***p-value < 0.001.

As far as the differences among the vineyards are concerned, a significantly higher average content over the years for Old SE was observed for almost all chemical families of compounds. Lower average amounts for terpenoids and benzenoids over the years were noted for the Young SW vineyard.

Analysis of variance was carried out to globally examine the effects of the vintage (year) on single compounds, highlighting significant differences among the years for the most important compounds, especially when the results were expressed as μg/100 berries; 2021 registered the higher amounts of the representative compounds mentioned before, except for the 3-oxo-α-ionol among norisoprenoids and for the C_6_ aldehydes and alcohols (**Supplementary Table 2**). In the vintage year 2020, the lowest amounts for almost all compounds were reported. Similar considerations can be made when the results are expressed as μg/kg of berries.

As regards the differences among vineyards, the concentrations of the predominant compounds of benzenoids and terpenoids were significantly higher in the Old SE grapes merely when the results were expressed as μg/100 berries, whereas no significant differences were noted for 3-oxo-α-ionol and 3-hydroxy-7,8-dihydro-β-ionol (see **Supplementary Tables 2**, **3**).

### 3.4 Aroma precursor trends in the vineyards

ANOVA results reported in **Table 4** show the mean differences between the vineyards calculated separately for each season. Variability was mostly associated with compounds such as terpenoids and especially benzenoids. Examining the differences between the vineyards in 2019, it can be noted that vineyard Old SE tends to show greater contents for all chemical classes of compounds, but statistically significant differences are highlighted only for benzenoid compounds.

**Table 4:** Average values of each aroma chemical group, berry weight, and sugar content in degrees Brix (°Brix) measured in the grapes of the three vineyards (O. SW: Old SW, O. SE: Old SE, and Y. SE: Young SW) for each year (2019, 2020, 2021, and 2022).

Year		2019				2020				2021				2022			
Vineyard		O. SW	O. SE	Y. SW	Sig.	O. SW	O. SE	Y. SW	Sig.	O. SW	O. SE	Y. SW	Sig.	O. SW	O. SE	Y. SW	Sig.
**Terpenoids**	μg/100 berries	56	99	34	ns	50	63	46	ns	109	162	63.7	ns	65 **b**	96 **a**	54 **b**	*
	μg/kg	474	656	261	ns	321	355	281	ns	653	779	468	ns	569 **b**	673 **a**	574 **b**	***
**C_13_-Norisoprenoids**	μg/100 berries	25	40	16	ns	36	33	26	ns	41	69	39	ns	36	57	33	ns
	μg/kg	206	262	123	ns	231	188	161	ns	248	330	273	ns	317	402	380	ns
**Benzenoids**	μg/100 berries	50 **b**	149 **a**	54 **b**	***	56	57	50	ns	168 **a**	148 **a**	38.4 **b**	**	128	161	102	ns
	μg/kg	422 **b**	988 **a**	409 **b**	**	359	324	306	ns	1.009 **a**	710 **a**	270 **b**	**	1.123	1.132	1.117	ns
**C_6_ aldehydes and alcohols**	μg/100 berries	11	13	10	ns	12	18	14	ns	15 **b**	24 **a**	5 **c**	**	18	20	14	ns
	μg/kg	96	88	75	ns	79	100	86	ns	88 **a**	116 **a**	32 **b**	**	163	148	151	ns
**Berry weight**	g	1.2 **b**	1.5 **a**	1.3 **b**	*	1.6	1.9	1.9	ns	1.7	1.8	1.7	ns	1.1 **b**	1.5 **a**	0.9 **c**	***
**Total soluble solids**	°Brix	26	25	25	ns	23 **b**	25 **a**	24 **a**	*	25	25	24	ns	27 **a**	27 **a**	25 **b**	**

Data were subjected to the analysis of variance (ANOVA) and post-hoc Tukey test. Means followed by a different letter are significantly different. ANOVA significance, ns (not significant): p-value > 0.05, ⁠*0.01 ≤ p-value < 0.05, ⁠**0.001 ≤ p-value < 0.01, ⁠***p-value < 0.001.

In 2020 vintage, no statistically significant differences were observed among vineyards but generally the old vineyards had higher contents of aroma precursors than the young one.

As in the previous vintage 2019, also in 2021, vineyard Old SE tends to show higher values for all chemical classes of compounds. Statistically significant differences are observed only regarding the concentrations of C_6_ aldehydes and alcohols and benzenoids. Generally, a higher concentration of terpenoids and benzenoids in the grapes of old-vine vineyards was noted with respect to Young SW grapes who showed the lowest values for all precursors and significantly lowest contents of benzenoids.

Finally, in 2022, grape berries of all the studied vineyards are characterized by high contents of benzenoids while Old SE grapes tend to contain higher amounts of all the groups of compounds as observed for the previous years but are significantly richer in terpenoids as well (per 100 berries and per kg of berries).

### 3.5 Vintage effect on aroma precursors

**Figure 2:**
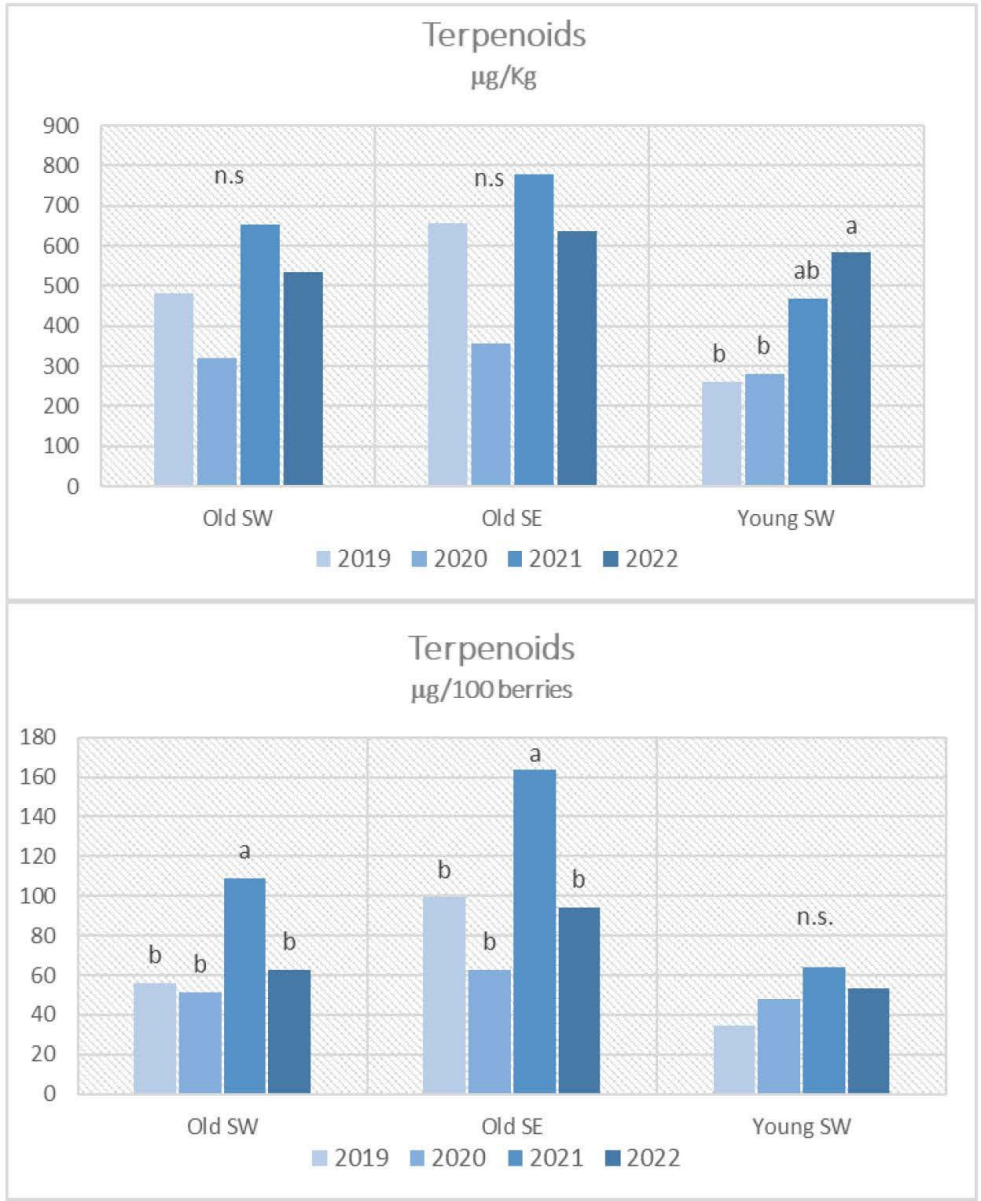
Terpenoids contents in Grignolino berries (µg/100 berries and µg/kg of berries) measured in each vintage year (2019, 2020, 2021, 2022) for the three vineyards (Old SW, Old SE, Young SE). Data were subjected to the analysis of variance (ANOVA) and post hoc Turkey test. Different letters indicate contents significantly different among the years for P<0.05, n.s.: not significant.

**Figure 3:**
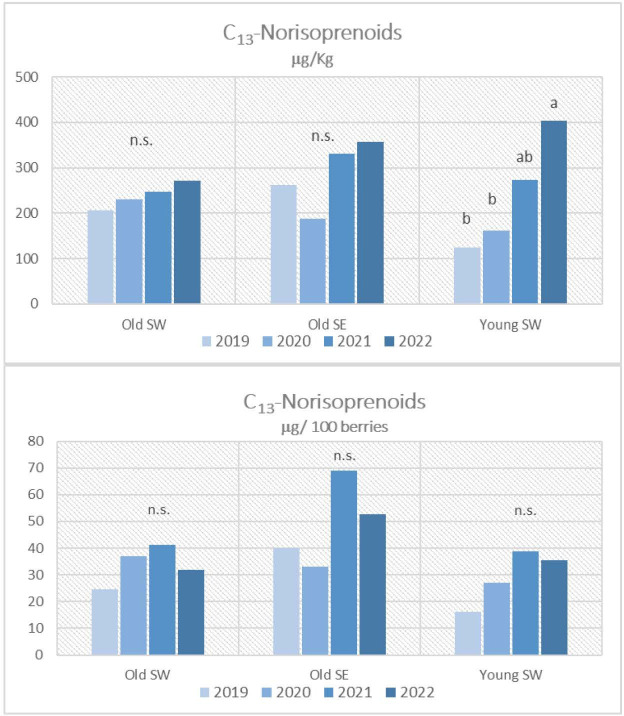
C_13_-norisoprenoid contents in Grignolino grape berries (µg/100 berries and µg/kg of berries) measured in each vintage year (2019, 2020, 2021, 2022) for the three vineyards (Old SW. Old SE, Young SE). Data were subjected to the analysis of variance (ANOVA) and *post hoc* Turkey test. Different letters indicate contents significantly different among the years for P<0.05. n.s.: not significant.

**Figure 4:**
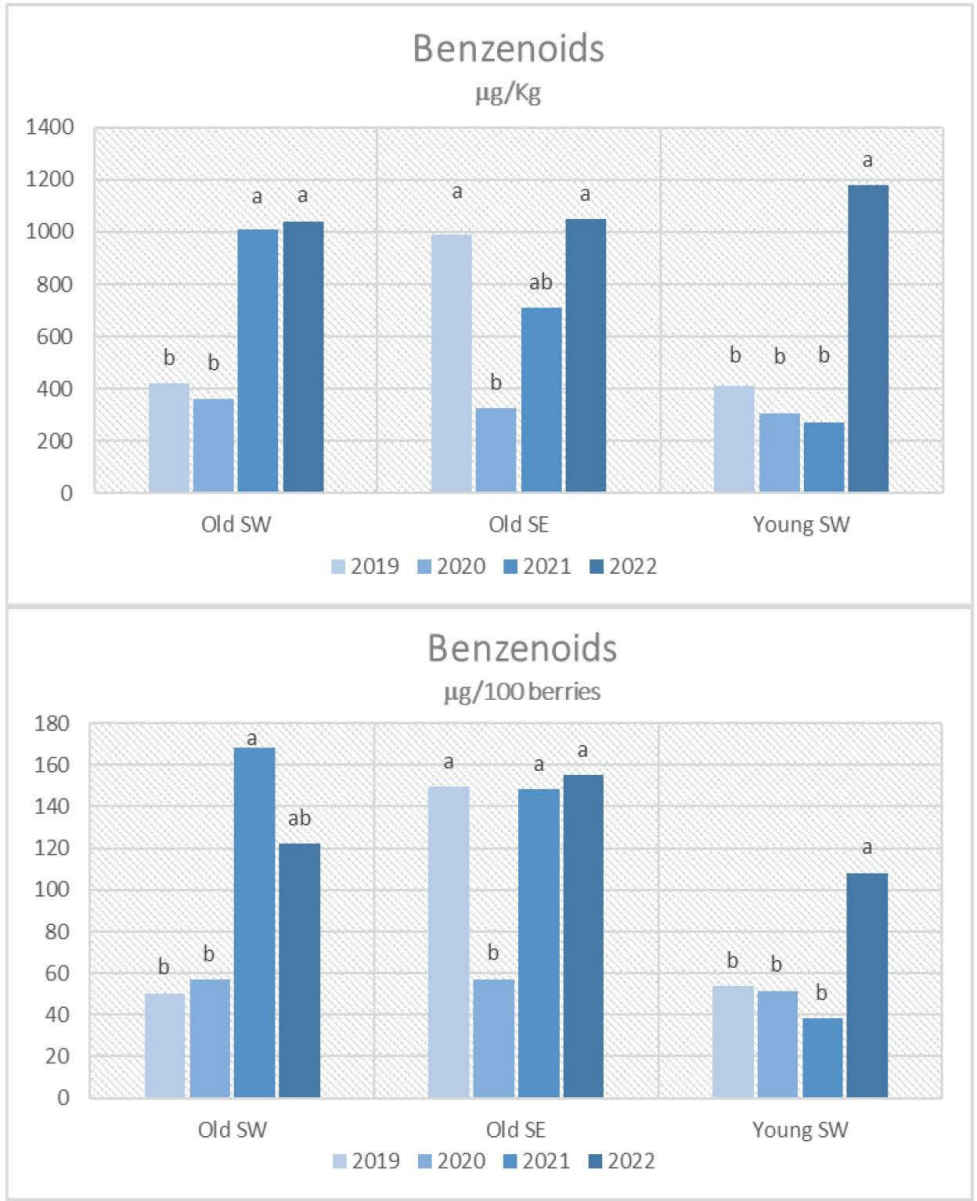
Benzenoids contents in Grignolino berries (µg/100 berries and µg/kg of berries) measured in each vintage year (2019, 2020, 2021, 2022) for the three vineyards (Old SW, Old SE, Young SE). Data were subjected to the analysis of variance (ANOVA) and post hoc Turkey test. Different letters indicate contents significantly different among the years for P<0.05. n.s.: not significant.

The mean differences for aroma precursors among the vintages, calculated for each vineyard, are highlighted in **Figures 2**–**4**. Meteorological conditions during 2021 favored the accumulation of significant amounts of terpenoids and benzenoids in the berries of the examined vineyards for the old ones, especially when results are expressed per berry. 2021 vintage tended to favor C_13_-norisoprenoids amounts too, but not in a significant way. Significantly higher values of C_13_-norisoprenoids were noticed in 2022 only for the Young SW vineyard when results are expressed per kg of berries. Benzenoids were the main compounds that accumulated in Grignolino berries especially during 2022. As far as vintage 2020 is concerned, the lowest concentrations for all classes of aroma precursors were reported especially for Old SE with respect to the previous years.

Although similar considerations can be made for old-vine vineyards, the effect of vintage instead was different for the young-vine vineyard (Young SW) which showed significantly higher amounts of almost all classes of precursors in 2022 when results were expressed in μg per kg due to the excessive low berry weight. The worst vintage year especially for terpenol and C_13_-norisoprenoid accumulation was 2019 for this vineyard.

### 3.6 PCA results

**Figure 5:**
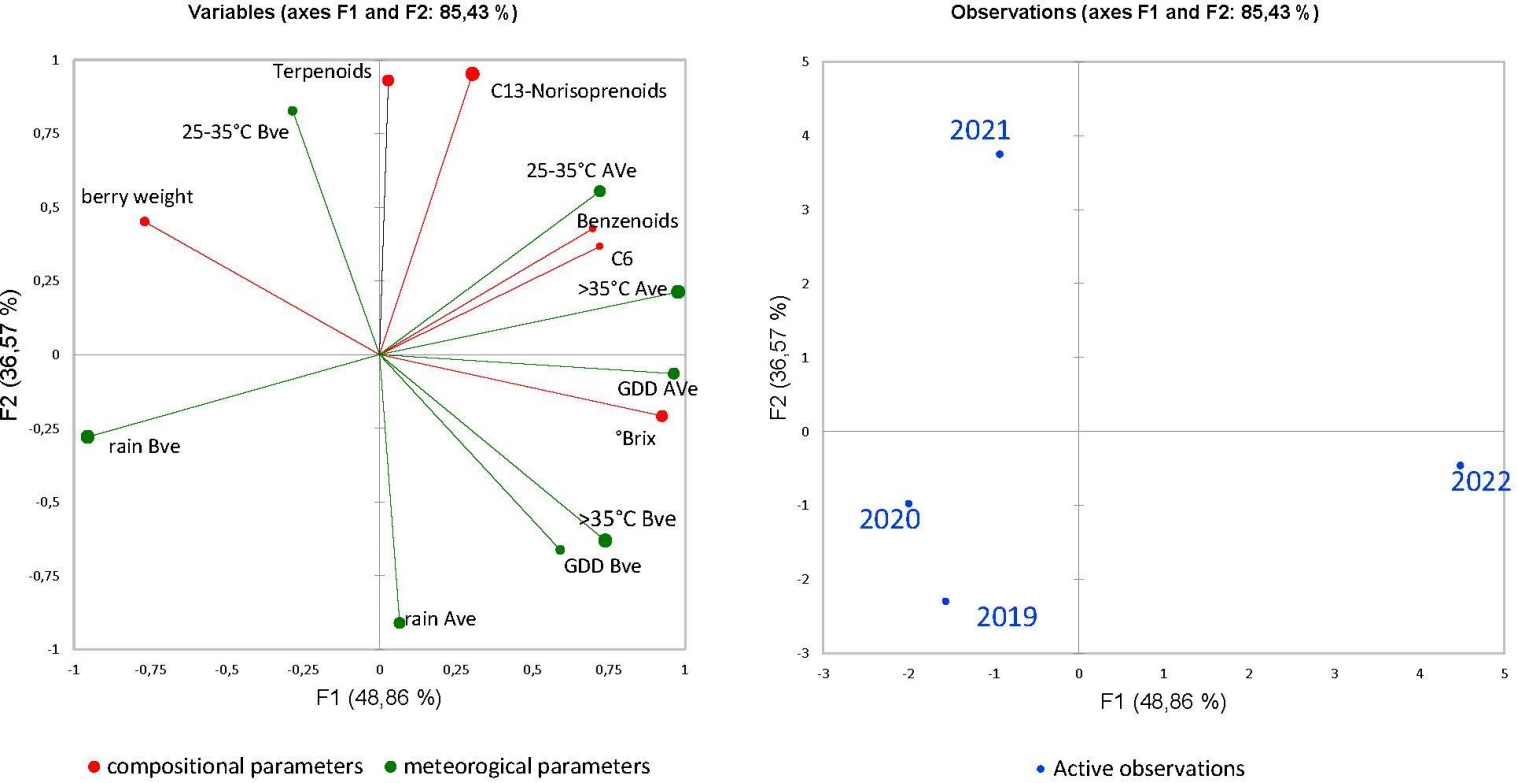
PCA of, °Brix, berry weight and meteorological conditions (BVe: before veraison and AVe: after veraison), in correlation with the classes of the aroma precursors expressed as µg /100 berries and the projection of the vintage years.

Variables related to the grape characteristics (berry weight and °Brix) and to aroma precursors (μg/100 berries) have been subjected to PCA in relation to the meteorological parameters. The PCA model explained an adequate level of variance. Climatic factors that characterized each year such as the days of optimal and maximum temperatures, GDD, and rain distribution before (BVe) and after veraison (AVe) were considered and indicated as 25–35°C BVe and AVe, >35° BVe and Ave, GDD BVe and AVe, and rainfall BVe and Ave in **Figure 5**.

Results (**Figure 5**) showed the projection of the year vintages and vineyards in the space defined by the meteorological parameters related to the first stage of berry development (from April until veraison) and to the second part of ripening (from veraison until harvest) together with berry weight, °Brix, and the concentration of the four groups of aroma precursors of the grapes at harvest, expressed as µg/100 berries (**Figure 5**). The two components (PC1 and PC2) explained 85.43% of the total variance. The first component (48.86% of total variance) is positively correlated with sugar content, benzenoid amounts, the GDD, and temperature ranges measured after veraison, and negatively correlated with the berry weight and the total days of rainfall calculated before veraison. The second component (36.57% of total variance) correlates positively with C_13_-norisoprenoids and terpenoids among aroma variables and the range 25–35°C before veraison but negatively with the rain after veraison. PCA results showed that each season was linked to a distinct group.

More specifically, PCA clearly emphasizes the positive relation between the amounts of C_13_-norisoprenoids and terpenoids in grapes and the total days of optimal temperature in the early stage of berry development (25–35°C BVe) and links season 2021 to these variables. Furthermore, a positive correlation of °Brix, the amounts in benzenoids, GDD, and hot days after veraison with 2022 is noted. The negative correlation of most compounds with rainfall both before and after veraison is evidenced mainly for terpenoids and C_13_-norisoprenoids, and the projection of seasons 2019 and 2020 follows the same pattern. Optimal conditions like those during 2021 seem to emphasize the differences among vineyards that otherwise are flattened as observed for instance for vintage 2020 (**Table 4**).

## 4 Discussion

The observations of this multi-year research study have permitted us to draw some important conclusions. Grapes harvested over the 4 years of study in the three vineyards reached similar physical and chemical parameters at maturity, showing instead differences for °Brix in 2022 and polyphenolic content in 2020. The meteorological pattern observed for each vintage influenced the size and the composition of the berries. The hot (>30°C) and dry pre-veraison period (June) observed in 2019 has probably penalized the berry weight at harvest. Despite the increased skin-to-pulp ratio in 2019, terpenol and norisoprenoid amounts were lower especially for the Young SW grapes probably due to a high rainfall just before veraison that alternated with high temperature (>35°C) (**Figure 1A**) and to the low GDD in the post-veraison stage. According to some literature, the most important terpenes seem to be negatively correlated with high precipitations before veraison (Rienth et al., 2021). Furthermore, most water-involved studies on grape aroma compounds show very heterogeneous results, depending on the type of aroma compounds considered and certainly on the water retention capacity of the soil. However, the reported effects of water availability on aroma compounds are less evident than for phenolic compounds (Alem et al., 2019).

The low concentrations for all classes of aroma precursors reported in 2020 can be attributed to the abundant rainfall close to harvest time that affected their contents in the berry. Rainfall concentrated in the period of berry development and ripening, especially before veraison and close to harvest, has also flattened the differences among vineyards. It is remarkable that 2020 presents the lowest content of precursors especially in terpenoids and benzenoids even if the same average weight of berry was registered at harvest as for the year 2021.

Mild spring temperature conditions and a regular distribution of rainfall during the green phase as well as a warm (25–35°C) but not hot and dry post-veraison period until harvest that characterized 2021 vintage favored the accumulation of all classes of aroma precursors but especially terpenoids in the berries of old vineyards.

The meteorological conditions that occurred during 2022 affected vine behavior by anticipating the timing of the phenological phases; veraison and harvest dates occurred earlier than in the previous years. GDD and hot days after veraison also characterized this season, highlighting a positive correlation with the higher °Brix and the amounts in benzenoids. Intense conditions occurred in terms of high temperature in combination with probably less water due to the lack of rainfall in the pre-veraison stage, also attributed to, as in 2019, a significantly lower berry weight especially for the grapes produced by the young vines, which also accumulated less sugar. According to previous research, water deficit stress during the green growth phase has the highest impact on final berry volume (He et al., 2020; Rienth et al., 2021). Furthermore, high amounts of benzenoids were reported in the grapes of all vineyards, especially in the Young SW grapes, where higher amounts were found with respect to the previous years. According to literature, water deficit can cause a reduction of berry volume and an increase of the skin-to-pulp ratio, leading to a higher concentration of these compounds synthesized in the skin cells. Environmental stresses, indeed, can activate the shikimate acid pathway and further promote the accumulation of secondary metabolites such as benzenoids that share with flavonoids the same substrate phenylalanine (Zhang et al., 2012).

As regards the differences noted among the vineyards, optimal conditions seem to emphasize the differences among vineyards as highlighted by the PCA (**Figure 5B**). The younger-vine vineyard showed a different compositional variability among the vintages, mainly as regards the effect of the vintage on the berry weight, which tends to show lower concentrations in aroma precursors. The grapes produced from the old southeastern vineyard instead showed higher amounts for almost all chemical classes and in each season. Under dry–hot conditions like those verified during 2022, grape cluster exposure to sunlight tends to raise both solar radiation and daytime temperature on grapes, and gives rise to the phenomenon of degradation of aroma compounds (Scafidi et al., 2013; Asproudi et al., 2016). Considering that, in the Northern Hemisphere, south- and west-facing areas are warmer, characterized by the harsh afternoon sun, such conditions, more than the eastern one, can stress vines during ripening in warm climates. Old SE grapes presented significantly higher amounts of terpenoids in 2022, showing that the cooler and fresh seasonal conditions during ripening may protect the precursors that accumulated in the grapes during the earlier stages of ripening (Young et al., 2016).

This research study highlighted that the current climate change conditions in a hilly environment may positively affect the quality of Grignolino grapes both by limiting the differences on bunch ripening and by promoting the accumulation of secondary metabolites, especially in the older vineyards. Furthermore, new knowledge was added on how specific meteorological conditions such as rainfall and temperature patterns and their timing during berry development and ripening may impact grape aroma precursors, to adapt new strategies aimed at maintaining high-quality production.

## Data availability statement

The original contributions presented in the study are included in the article/**Supplementary Material**. Further inquiries can be directed to the corresponding author.

## Author contributions

AA and MP devised the main body, structure, and research content of the manuscript. AA, FB, and VR provided laboratory and instrumental analyses. SG provided assistance as regards experimental plan, sample collection, and vineyard information. All authors reviewed the manuscript. All authors contributed to the article and approved the submitted version.
