# Supplementary material for: Aroma precursors of Grignolino grapes (Vitis vinifera L.) and their modulation by vintage in a climate change scenario
Source: Front Plant Sci. 2023 Aug 4;14:1179111. doi: 10.3389/fpls.2023.1179111 (PMC10436553; doi:10.3389/fpls.2023.1179111)
Supplement: Supplementary file 1 [file Table_1.docx]

| **LRI Lit ^1^** | **LRI Cal. ^2^** | **Ref.^3^** | **Gr ^4^** | **Name** | **IUPAC Name** |
| --- | --- | --- | --- | --- | --- |
| 1196-1238 | 1233 | a | C6 | trans-2-hexenal | (E)-hex-2-enal |
| 1316-1377 | 1359 | a | C6 | 1-hexanol | hexan-1-ol |
| 1344-1399 | 1371 | a | C6 | trans-3-hexen-1-ol | (E)-hex-3-en-1-ol |
| 1351-1405 | 1392 | a | C6 | cis-3-hexen-1-ol | (Z)-hex-3-en-1-ol |
| 1377-1419 | 1413 | a | C6 | trans-2-hexen-1-ol | (E)-hex-2-en-1-ol |
| 1410-1478 | 1448 | a | T | cis-furan linalool oxide | 2-(5-ethenyl-5-methyloxolan-2-yl)propan-2-ol |
| 1429-1481 | 1476 | a | T | trans-furan-linalool oxide | 2-(5-ethenyl-5-methyloxolan-2-yl)propan-2-ol |
| 1481-1555 | 1532 | a | B | benzaldehyde | benzaldehyde |
| 1507-1564 | 1553 | a | T | linalool | 3,7-dimethylocta-1,6-dien-3-ol |
| 1580-1616 | 1619 | a | T | hotrienol | 3,7-Dimethyl-1,5,7-octatrien-3-ol |
| 1629** | 1621 | b | T | p-Menth-1-en-9-al I | 2-(4-methylcyclohex-3-en-1-yl)propanal |
| 1632** | 1624 | b | T | p-Menth-1-en-9-al II | 2-(4-methylcyclohex-3-en-1-yl)propanal |
| 1659-1724 | 1702 | a | T | α-terpineol | 2-[4-methylcyclohex-3-en-1-yl]propan-2-ol |
| 1680-1750 | 1743 | a | T | geranial | (2E)-3,7-dimethylocta-2,6-dienal |
| 1698-1778 | 1746 | c | T | trans-pyran Linalool oxide | (3R,6S)-6-ethenyl-2,2,6-trimethyloxan-3-ol |
| - | - | - | N | U.N 1 (138; 193; 208) | - |
| 1708-1772 | 1770 | c | T | cis-pyran Linalool oxide | (3S,6S)-6-ethenyl-2,2,6-trimethyloxan-3-ol |
| 1734-1789 | 1775 | a | T | citronellol | 3,7-dimethyloct-6-en-1-ol |
| 1727-1809 | 1782 | a | B | methyl salicylate | methyl 2-hydroxybenzoate |
| 1752-1832 | 1809 | a | T | nerol | (2Z)-3,7-dimethylocta-2,6-dien-1-ol |
| 1795-1865 | 1857 | a | T | geraniol | (2E)-3,7-dimethylocta-2,6-dien-1-ol |
| 1807-1873 | 1858 | a | C6 | hexanoic acid | hexanoic acid |
| nf | 1866 | - | T | 2-hydroxy-1,8-cineole | (1R)-1,3,3-trimethyl-2-oxabicyclo[2.2.2]octan-6-ol |
| 1821-1905 | 1888 | a | B | benzyl alcohol | phenylmethanol |
| 1859-1944 | 1923 | a | B | 2-phenylethanol | 2-phenylethanol |
| 1914-1963 | 1955 | c | T | terpendiol I | 3,7-dimethylocta-1,5-dien-3,7-diol |
| 1933-2032 | 2020 | a | B | phenol | phenol |
| 2100-2198 | 2179 | a | B | eugenol | 2-methoxy-4-prop-2-enylphenol |
| 2145-2219 | 2209 | a | B | 4-vinylguaiacol | 4-ethenyl-2-methoxyphenol |
| n.a. | 2220 | - | T | hydroxycitronellol | 3,7-dimethyloctane-1,7-diol |
| 2251 | 2246 | h | T | 8-hydroxylinalool | (2E)-2,6-dimethylocta-2,7-diene-1,6-diol |
| 2220 | 2249 | c | T | 8-hydroxy-6,7-dihydrolinalool | 3,7-dimethyl-6-octen-3-ol |
| 2266-2298 | 2287 | c | T | trans-8-hydroxylinalool | 2,7-octadiene-1,6-diol, 2,6-dimethyl-, (E)- |
| 2316-2343 | 2328 | c | T | cis-8-hydroxylinalool | 2,7-octadiene-1,6-diol, 2,6-dimethyl-, (Z)- |
| 2347 | 2357 | d | T | geranic acid | (2E)-3,7-dimethylocta-2,6-dienoic acid |
| 2330 | 2365 | e | B | isoeugenol | 2-methoxy-4-[(*E*)-prop-1-enyl]phenol |
| 2357-2420 | 2415 | c | B | 4-vinylphenol | 4-ethenylphenol |
| 2418 | 2456 | f | N | 3,4-dihydro-3-oxoactinidol I | 2-(1-hydroxyethyl) - 4,4,7a-trimethyl-2,5,6,7-tetrahydro-1-benzofuran-6-ol |
| 2456 | 2487 | f | N | 3,4-dihydro-3-oxoactinidol II | 2-(1-hydroxyethyl) - 4,4,7a-trimethyl-2,5,6,7-tetrahydro-1-benzofuran-6-ol |
| 2476 | 2491 | f | N | 3,4-dihydro-3-oxoactinidol III | 2-(1-hydroxyethyl) - 4,4,7a-trimethyl-2,5,6,7-tetrahydro-1-benzofuran-6-ol |
| 2538 | 2546 | c | T | p-ment-1-en-7,8-diol | 2-[(1R)-4-(hydroxymethyl)cyclohex-3-en-1-yl]propan-2-ol |
| 2504-2563 | 2558 | c | N | 3-hydroxy-β-damascone | (E)-1-(3-hydroxy-2,6,6-trimethylcyclohexen-1-yl)but-2-en-1-one |
| 2571 | nf | i | N | 3,4-didehydro-β-ionone | 4-(2,6,6-Trimethylcyclohexa-1,3-dienyl)but-3-en-2-one |
| 2531-2605 | 2590 | c | B | vanillin | 4-hydroxy-3-methoxybenzaldehyde |
| 2565-2635 | 2627 | c | B | methylvanillate | methyl 4-hydroxy-3-methoxybenzoate |
| 2639 | 2656 | c | N | 3-oxo−α-ionol | 4-[(E)-3-hydroxybut-1-enyl]-3,5,5-trimethylcyclohex-2-en-1-one |
| 2640 | 2661 | c | N | 4-oxo-β-ionol | 3-[(E)-3-hydroxybut-1-enyl]-2,2,4-trimethylcyclohex-3-en-1-one |
| 2626-2667 | 2663 | a | B | acetovanillone | 1-(4-hydroxy-3-methoxyphenyl)ethanone |
| **-** | 2683 | - | N | U.N. 2 (121-136-161) | - |
| 2726 | 2774 | i | N | 3-hydroxy-7,8-dihydro-β-ionol | 4-(3-hydroxybutyl)-3,5,5-trimethylcyclohex-3-en-1-ol |
| 2800 | 2787 | c | N | 2,3-dehydro-4-oxo-β-ionol | 3-[(*E*)-3-hydroxybut-1-enyl]-2,4,4-trimethylcyclohexa-2,5-dien-1-one |
| 2829 | 2823 | c | B | Zingerone | 4-(4-hydroxy-3-methoxyphenyl)butan-2-one |
| 2776 | 2855 | c | N | 3-hydroxy-5,6-epoxy-β-ionol | 6-[(E)-3-hydroxybut-1-enyl]-1,5,5-trimethyl-7-oxabicyclo[4.1.0]heptan-3-ol |
| 3099 | 2880 | c | B | homovanillic alohol | 2-(4-hydroxy-3-methoxyphenyl)acetic acid |
|  |  |  |  |  |  |
|  |  |  |  |  |  |

^1^**LRI Lit.**: Linear Retention Index values retrieved from literature. **^2^ LRI Cal.**: Linear Retention Index calculated comparing retention times of a homologous series of n-alkanes and analytes, separated with the used GC method. ^3^**Ref**.: The references from which LRI values were obtained from literature are listed below. ^4^**Gr**. Compounds are grouped following their chemical structure. C6 alcohols with a six-carbon skeleton (also known as leaf alcohols), T: terpenois, N: norisopenoids, B: benzenoids.

1. Babushok, V. I., Linstrom, P. J., and Zenkevich, I. G. (2011). Retention Indices for Frequently Reported Compounds of Plant Essential Oils. *Journal of Physical and Chemical Reference Data* 40, 043101. doi: 10.1063/1.3653552.
2. Genisheva, Z., and Oliveira, J. M. (2009). Monoterpenic Characterization of White Cultivars from Vinhos Verdes Appellation of Origin (North Portugal). Journal of the Institute of Brewing 115, 308–317. doi: 10.1002/j.2050-0416.2009.tb00386.x.
3. NIST Chemistry WebBook (n.d.). Available at: https://webbook.nist.gov/chemistry/ [Accessed December 29, 2021]
4. Soria, A. C., Sanz, J., and Martínez-Castro, I. (2009). SPME followed by GC–MS: a powerful technique for qualitative analysis of honey volatiles. Eur Food Res Technol 228, 579–590. doi: 10.1007/s00217-008-0966-z.
5. The Glycosidically Bound Volatile Compounds of Picea mariana (Mill.) B.S.P.: Journal of Essential Oil Research: Vol 6, No 1 (n.d.). Available at: https://www.tandfonline.com/doi/abs/10.1080/10412905.1994.9698323 [Accessed February 10, 2023].
6. Wirth, J., Guo, W., Baumes, R., and Günata, Z. (2001). Volatile Compounds Released by Enzymatic Hydrolysis of Glycoconjugates of Leaves and Grape Berries from Vitis vinifera Muscat of Alexandria and Shiraz Cultivars. *J. Agric. Food Chem.* 49, 2917–2923. doi: 10.1021/jf001398l.
