# Supplementary material for: Aroma precursors of Grignolino grapes (Vitis vinifera L.) and their modulation by vintage in a climate change scenario
Source: Front Plant Sci. 2023 Aug 4;14:1179111. doi: 10.3389/fpls.2023.1179111 (PMC10436553; doi:10.3389/fpls.2023.1179111)
Supplement: Supplementary file 2 [file Table_2.docx]

| Compounds | Year | | | | | Vineyard | | | |
| --- | --- | --- | --- | --- | --- | --- | --- | --- | --- |
|  | **2021** | **2022** | **2019** | **2020** | **Sig.** | **Old SE** | **Old SW** | **Young SW** | **Sig.** |
| 1-hexanol | 13,49 | 13,18 | 10,84 | 13,98 | ns | 16,7 **a** | 11,6 **b** | 10,3 **b** | ** |
| cis-3-hexenol | 0,43 | 0,59 | 0,23 | 0,31 | ns | 0,47 | 0,29 | 0,40 | ns |
| trans-2-hexenol | 0,23 | 0,14 | 0,1 | 0,1 | ns | 0,20 | 0,14 | 0,08 | ns |
| trans-furan linalool oxide | 1,24 **a** | 0,51 **b** | 0,421 **b** | 0,489 **b** | ** | 1,114 **a** | 0,488 **b** | 0,39 **b** | ** |
| cis-furan-linalool oxide | 2,49 **a** | 1,06 **b** | 0,933 **b** | 0,791 **b** | *** | 1,73 **a** | 1,31 **ab** | 0,92 **b** | * |
| Benzaldehyde | 0,25 **a** | 0,19 **ab** | 0,070 **bc** | 0,057 **c** | ** | 0,13 | 0,18 | 0,12 | ns |
| linalool | 0,04 | 0,09 | 0,09 | 0,09 | ns | 0,07 | 0,09 | 0,08 | ns |
| hotrienol | 0,27 **a** | 0,01 **b** | 0,17 **a** | 0,05 **b** | *** | 0,192 **a** | 0,12 **ab** | 0,07 **b** | ** |
| p-Menth-1-en-9-al (isomer I) | 0,02 **a** | 0,01 **b** | 0,01 **b** | 0,01 **b** | *** | 0,02 **a** | 0,01 **b** | 0,01 **b** | ** |
| p-Menth-1-en-9-al (isomer II) | 0,02 **a** | 0,01 **ab** | 0,01 **ab** | 0,006 **b** | * | 0,02 **a** | 0,01 **ab** | 0,01 **b** | * |
| a-terpineol | 4,17 **a** | 1,25 **b** | 1,314 **b** | 0,000 **b** | *** | 2,19 | 1,44 | 1,42 | ns |
| geraniale | 0,61 **a** | 0,01 **b** | 0,01 **b** | 0,05 **b** | *** | 0,24 **a** | 0,23 **a** | 0,04 **b** | ** |
| citronellolo | 1,09 **a** | 0,15 **b** | 0,49 **ab** | 0,35 **b** | * | 0,66 **a** | 0,43 **a** | 0,47 **a** | ns |
| trans-pyran Linalool oxide | 4,08 **a** | 1,48 **b** | 1,13 **b** | 1,34 **b** | *** | 3,58 **a** | 1,49 **b** | 0,94 **b** | *** |
| metilsalicilato | 0,80 **b** | 1,66 **a** | 0,55 **b** | 0,62 **b** | ** | 1,88 **a** | 0,44 **b** | 0,41 **b** | *** |
| cis-pyran Linalool oxide | 1,21 | 0,62 | 0,18 | 0,71 | ns | 1,06 | 0,48 | 0,51 | ns |
| nerol | 4,48 **a** | 2,06 **b** | 1,997 **b** | 2,14 **b** | *** | 3,56 **a** | 2,59 **ab** | 1,86 **b** | ** |
| geraniol | 15,5 **a** | 6,96 **b** | 5,74 **b** | 8,37 **b** | *** | 11,09 **a** | 9,52 **a** | 6,78 **b** | ** |
| benzyl alcohol | 44,4 **a** | 43,5 **ab** | 33,7 **ab** | 23,3 **b** | * | 48,1 **a** | 36,8 **ab** | 23,7 **b** | ** |
| phenylethyl alcohol | 25,7 **ab** | 31,74 **a** | 18,16 **b** | 14,41 **b** | ** | 26,5 **a** | 24,7 **ab** | 16,3 **b** | * |
| Terpendiol I | 5,83 **a** | 1,54 **b** | 4,08 **a** | 1,702 **b** | *** | 6,18 **a** | 2,32 **b** | 1,36 **b** | *** |
| eugenol | 0,95 | 0,89 | 0,35 | 0,322 | ns | 0,97 **a** | 0,68 **ab** | 0,24 **b** | * |
| hydroxycitronellolo | 3,17 **a** | 1,94 **ab** | 1,33 **bc** | 0,417 **c** | *** | 2,27 | 1,52 | 1,36 | ns |
| 4-vinylguaiacol | 7,65 **a** | 5,85 **b** | 5,76 **b** | 2,692 **c** | *** | 8,56 **a** | 4,82 **b** | 3,09 **c** | *** |
| trans-8-hydroxylinalool | 4,89 **a** | 4,50 **a** | 1,40 **b** | 2,178 **b** | *** | 4,24 **a** | 3,11 **ab** | 2,38 **b** | * |
| cis-8-hydroxylinalool | 20,1 **a** | 12,64 **b** | 11,25 **b** | 11,484 **b** | * | 21,78 **a** | 12,5 **b** | 7,34 **b** | *** |
| geranic acid | 19,9 **a** | 13,08 **b** | 10,25 **b** | 10,963 **b** | *** | 16,15 **a** | 12,4 **b** | 12,04 **b** | * |
| isoeugenol | 0,47 **a** | 0,82 **a** | 6,30 **a** | 0,462 **a** | ns | 4,97 **a** | 0,50 **a** | 0,57 **a** | ns |
| 3,4-dihydro-3-oxoactinidol I | 1,82 **a** | 0,35 **b** | 0,14 **b** | 0,086 **b** | *** | 0,61 | 0,52 | 0,66 | ns |
| 3,4-dihydro-3-oxoactinidol II | 2,34 **a** | 1,47 **b** | 0,97 **b** | 1,197 **b** | *** | 2,24 **a** | 1,21 **b** | 1,03 **b** | *** |
| 3,4-dihydro-3-oxoactinidol III | 2,26 | 1,94 | 1,49 | 1,188 | ns | 2,73 **a** | 1,22 **b** | 1,21 **b** | ** |
| 4-vinylphenol | 15,6 **a** | 1,97 **c** | 6,35 **b** | 1,769 **c** | *** | 7,74 **a** | 7,23 **ab** | 4,31 **b** | * |
| p-ment-1-ene-7,8-diolo | 24,3 **a** | 18,4 **ab** | 20,4 **ab** | 11,1 **b** | * | 24,4 **a** | 17,5 **ab** | 13,86 **b** | * |
| 3-OH-b-damascone | 9,06 **a** | 6,70 **ab** | 4,93 **b** | 6,23 **ab** | * | 8,77 **a** | 6,27 **ab** | 5,16 **b** | ** |
| vanillin | 2,60 | 2,28 | 1,87 | 1,281 | ns | 2,81 **a** | 1,87 **ab** | 1,35 **b** | ** |
| methylvanillate | 9,08 **ab** | 14,1 **a** | 4,39 **b** | 3,15 **b** | ** | 10,4 **a** | 6,58 **a** | 6,05 **a** | ns |
| 4-oxo-b-ionolo | 1,32 **a** | 1,00 **b** | 1,29 **a** | 1,221 **a** | *** | 1,33 **a** | 0,97 **ab** | 0,57 **b** | ** |
| acetovanillone | 4,81 **a** | 4,09 **a** | 1,68 **b** | 1,05 **b** | *** | 3,56 | 2,46 | 1,91 | ns |
| 3-oxo-a-ionol | 13,66 | 11,35 | 8,19 | 12,38 | ns | 13,97 | 10,72 | 9,49 | ns |
| Norisoprenoid (121-136-161) | 4,76 **a** | 2,15 **b** | 1,20 **b** | 1,33 **b** | *** | 4,04 **a** | 1,42 **b** | 1,62 **b** | ** |
| 3-hydroxy-7,8-dihydro-b-ionol | 6,28 **ab** | 7,13 **a** | 4,50 **b** | 4,29 **b** | * | 6,35 **a** | 5,32 **a** | 4,97 **a** | ns |
| Zingerone | 2,99 | 2,98 | 4,06 | 2,94 | ns | 4,33 **a** | 3,03 **b** | 2,38 **b** | ** |
| 3-hydroxy-5,6-epoxy-b-ionol | 2,46 | 0,97 | 0,94 | 0,42 | ns | 1,89 | 1,00 | 0,69 | ns |
| homovanillic alohol | 2,40 | 2,74 | 0,93 | 2,48 | ns | 2,68 | 2,04 | 1,69 | ns |

**Table 2**. Average values of vol**a**tile compounds (μg/100 **b**erries) in the grapes of the three vineyards (Old SW, Old SE, Young SW) for each vintage year (2019, 2020, 2021, 2022). Data were subjected to the analysis of variance (ANOVA) and *post hoc* Tukey test. **A**NOV**A** signifi**ca**n**c**e, ns (not signifi**ca**nt): p-v**a**lue>0.05, ⁠*: 0.01≤p-v**a**lue<0.05, ⁠**: 0.001≤p-v**a**lue<0.01, ⁠***: p-v**a**lue<0.001; Different letters indi**ca**te different le**a**st squ**a**re me**a**ns.
