# Supplementary material for: Aroma precursors of Grignolino grapes (Vitis vinifera L.) and their modulation by vintage in a climate change scenario
Source: Front Plant Sci. 2023 Aug 4;14:1179111. doi: 10.3389/fpls.2023.1179111 (PMC10436553; doi:10.3389/fpls.2023.1179111)
Supplement: Supplementary file 3 [file Table_3.docx]

| Compounds | Year | | | | | Vineyard | | | |
| --- | --- | --- | --- | --- | --- | --- | --- | --- | --- |
|  | **2021** | **2022** | **2019** | **2020** | Sig. | **Old SE** | **Old SW** | **Young SW** | Sig. |
| 1-hexanol | 73,3 | 118 | 81,3 | 85,2 | ns | 99,48 | 84,7 | 84,7 | ns |
| cis-3-hexenol | 2,33 **ab** | 5,63 **a** | 1,78 **b** | 1,87 **b** | * | 2,82 | 2,19 | 3,69 | ns |
| trans-2-hexenol | 1,26 | 1,21 | 0,70 | 0,58 | ns | 1,18 | 0,99 | 0,63 | ns |
| trans-furan linalool oxide | 6,89 **a** | 4,09 **ab** | 2,99 **b** | 3,00 **b** | ** | 6,51 **a** | 3,32 **b** | 2,91 **b** | ** |
| cis-furan-linalool oxide | 14,3 **a** | 9,12 **ab** | 7,08 **b** | 4,93 **b** | ** | 10,1 | 9,11 | 7,29 | ns |
| Benzaldehyde | 1,50 **a** | 1,74 **a** | 0,55 **b** | 0,35 **b** | *** | 0,77 | 1,29 | 1,00 | ns |
| linalool | 0,23 | 0,81 | 0,63 | 0,60 | ns | 0,43 | 0,68 | 0,59 | ns |
| hotrienol | 1,52 **a** | 0,13 **b** | 1,22 **a** | 0,32 **b** | *** | 1,09 | 0,79 | 0,50 | ns |
| p-Menth-1-en-9-al (isomer I) | 0,12 **a** | 0,08 **ab** | 0,04 **b** | 0,03 **b** | *** | 0,1 **a** | 0,06 **ab** | 0,04 **b** | * |
| p-Menth-1-en-9-al (isomer II) | 0,10 | 0,09 | 0,06 | 0,04 | ns | 0,09 **a** | 0,08 **ab** | 0,04 **b** | * |
| a-terpineol | 24,3 **a** | 10,7 **b** | 9,88 **b** | 8,90 **b** | ** | 12,4 | 10,3 | 10,9 | ns |
| geraniale | 3,37 **a** | 0,10 **b** | 0,10 **b** | 0,33 **b** | ** | 1,16 | 1,38 | 0,31 | ns |
| citronellolo | 6,43 **a** | 1,13 **b** | 3,59 **ab** | 2,17 **b** | * | 3,82 | 2,89 | 3,27 | ns |
| trans-pyran Linalool oxide | 22,1 **a** | 12,2 **b** | 8,16 **b** | 8,17 **b** | *** | 20,5 **a** | 10,1 **b** | 7,36 **b** | *** |
| metilsalicilato | 4,347 **a** | 13,3 **a** | 3,80 **a** | 3,77 **a** | * | 12,5 **a** | 2,91 **b** | 3,46 **b** | ** |
| cis-pyran Linalool oxide | 6,20 | 5,32 | 1,39 | 4,34 | ns | 5,94 | 3,28 | 3,72 | ns |
| nerol | 25,4 **a** | 17,6 **ab** | 14,8 **b** | 13,1 **b** | ** | 20,9 **a** | 18,1 **ab** | 14,1 **b** | * |
| geraniol | 89,2 **a** | 60,2 **b** | 42,5 **b** | 51,4 **b** | *** | 64,6 | 66,8 | 51,1 | ns |
| benzyl alcohol | 249 ab | 385 a | 240 ab | 143 b | * | 300 | 258 | 204 | ns |
| phenylethyl alcohol | 144 b | 282 a | 133 b | 89,3 **b** | *** | 166 | 178 | 142 | ns |
| Terpendiol I | 31,5 **a** | 12,6 **b** | 28,8 **a** | 10,0 **b** | ** | 36,2 **a** | 15,9 **b** | 10,0 **b** | *** |
| eugenol | 5,65 **a** | 6,92 | 2,59 | 1,95 | ns | 6,42 | 4,72 | 1,69 | ns |
| hydroxycitronellolo | 18,42 **a** | 16,96 **a** | 9,55 **b** | 2,57 **b** | *** | 13,7**a** | 10,7 | 11,2 | ns |
| 4-vinylguaiacol | 42,0 **a** | 50,3 **a** | 40,5 **ab** | 16,4 **b** | ** | 52,6 **a** | 33,3 **ab** | 26,0 **b** | ** |
| trans-8-hydroxylinalool | 27,2 **ab** | 40,3 **a** | 16,6 **b** | 13,3 **b** | *** | 27,9 | 24,1 | 20,9 | ns |
| cis-8-hydroxylinalool | 105 | 99,6 | 82.0 | 69,4 | ns | 126 a | 83,9 **b** | 56,4 **b** | *** |
| geranic acid | 116 **ab** | 117 a | 76.0 **b**c | 67,6 **c** | ** | 95,4 | 90,7 | 96,1 | ns |
| isoeugenol | 2,84 | 7,25 | 41,4 | 2,78 | ns | 32,1 | 3,88 | 4,74 | ns |
| 3,4-dihydro-3-oxoactinidol I | 10,4 **a** | 3,79 **b** | 1,15 **b** | 0,52 **b** | *** | 2,91 | 3,36 | 5,58 | ns |
| 3,4-dihydro-3-oxoactinidol II | 12,7 | 13,01 | 7,47 | 7,40 | ns | 13,0 | 8,63 | 8,73 | ns |
| 3,4-dihydro-3-oxoactinidol III | 12,4 | 16,7 | 10,8 | 7,32 | ns | 16,6 **a** | 8,66 **a** | 10,1 **a** | * |
| 4-vinylphenol | 88,1 **a** | 16,5 **b** | 46,4 **ab** | 10,7 **b** | ** | 43,8 | 44,6 | 32,9 | ns |
| p-ment-1-ene-7,8-diolo | 125 | 166 | 120 | 68,2 | ns | 148 | 126 | 84,4 | ns |
| 3-OH-b-damascone | 51,7 | 59,1 | 35,8 | 38,1 | ns | 53,2 | 44,1 | 41,4 | ns |
| vanillin | 14,7 **ab** | 20,3 **a** | 13,5 **ab** | 7,79 **b** | * | 17,3 | 13,1 | 11,9 | ns |
| methylvanillate | 49,4 **b** | 127 a | 31,6 **b** | 19,3 **b** | *** | 64,1 | 49,3 | 57,5 | ns |
| 4-oxo-b-ionolo | 7,26 | 7,00 | 9,36 | 7,60 | ns | 7,58 **a** | 6,71 **ab** | 3,87 **b** | * |
| acetovanillone | 26,7 **ab** | 36,6 **a** | 12,3 **b**c | 10,0 c | *** | 21,16 | 18,1 | 17,5 | ns |
| 3-oxo-a-ionol | 77,1 | 101 | 61,1 | 76,8 | ns | 83,6 | 76,5 | 77,4 | ns |
| Norisoprenoid (121-136-161) | 25,8 **a** | 18,7 **ab** | 8,53 **b** | 8,14 **b** | ** | 22,9 **a** | 9,72 **b** | 13,2 **ab** | ** |
| 3-hydroxy-7,8-dihydro-b-ionol | 36,8 **b** | 64,5 **a** | 33,1 **b** | 26,3 **b** | ** | 39,8 | 38,3 | 42,4 | ns |
| Zingerone | 16,6 | 26,8 | 29,6 | 18,0 | ns | 26,7 | 22,2 | 19,4 | ns |
| 3-hydroxy-5,6-epoxy-b-ionol | 12,6 | 9,36 | 6,83 | 2,57 | ns | 9,87 | 7,43 | 6,26 | ns |
| homovanillic alohol | 12,5 **ab** | 24,8 **a** | 7,42 **b** | 15,2 **ab** | * | 15,7 | 14,5 | 14,6 | ns |

**Table 3**. Average values of volatile compounds (μg/Kg of berries) n the grapes of the three vineyards (Old SW, Old SE, Young SW) for each vintage year (2019, 2020, 2021, 2022). Data were subjected to the analysis of variance (ANOVA) and *post hoc* Tukey test. ANOVA significance, ns (not significant): p-value>0.05, ⁠*: 0.01≤p-value<0.05, ⁠**: 0.001≤p-value<0.01, ⁠***: p-value<0.001; Different letters indicate different least square means.
